# Acute apnea and white blood cell count: A biphasic response formal comment on ‘Hematologic changes after short term hypoxia in non-elite apnea divers under voluntary dry apnea conditions’

**DOI:** 10.1371/journal.pone.0253584

**Published:** 2021-07-14

**Authors:** Janne Bouten, Jan G. Bourgois, Leen Lootens, Jan Boone

**Affiliations:** 1 Department of Movement and Sports Sciences, Ghent University, Ghent, Belgium; 2 Centre of Sports Medicine, Ghent University Hospital, Ghent, Belgium; 3 Doping Control Laboratory, Department of Diagnostic Sciences, Ghent University, Ghent, Belgium; The Ohio State University, UNITED STATES

Acute breath-holding poses a challenge to the human body as it deprives the body from its oxygen supply. In order to prevent the body against hypoxia, a series of physiological responses is elicited, called the diving response. This response is characterized by bradycardia, peripheral vasoconstriction and an increase in mean arterial pressure [1–4] and accompanied by a spleen contraction releasing red blood cells into circulation [5–7]. Although the impact of apnea induced splenic contraction on red blood cell related parameters (erythrocyte count, hemoglobin (Hb), hematocrit (Hct), reticulocytes) in the time span up to 20 minutes post apnea has been studied quite extensively, the effect on circulating white blood cells (WBC) [6] and the evolution of these parameters in the following hours is less known. In this context, the study ‘Hematologic changes after short term hypoxia in non-elite apnea divers under voluntary dry apnea conditions’ provides new insights revealing exhaustive data on all three blood cell lines up to four hours after acute static apnea.

Surprisingly however, the results of this study do not show an acute increase in Hct, [Hb] nor erythrocyte count, contradicting a well-established and documented apnea response. Based on experience with blood analysis in our lab, we expect that body position during [8], as well as activity before blood sampling might play a role [9]. In this context, it has been shown that walking as little as 50m already significantly increases [Hb] and Hct due to an acute decrease in plasma volume [9]. The methods section does not clarify how blood collection was performed nor mentions the activity and standardization in the time span before blood sample collection. It does however state that the apnea was performed in a horizontal position. In this case, the increase in [Hb] might very well be masked by an increase in plasma volume due to the prolonged time spent in a horizontal position while performing the breath-hold protocol. Indeed, changing from a standing to supine position has been shown to lead to an increase of around 418 mL of plasma volume (individually ranging from 149 to 717 mL) [10]. Supine body position resulted in lower values, i.e., a decrease of 2.3 and 7.1 g/L for [Hb] and 1.7 and 6.9% for Hct, compared to respective sitting and standing posture [8].

These findings emphasize the need for a standardized protocol prior to venous blood sampling in research in order to minimize pre-analytical variation. Therefore, the World Anti-Doping Agency (WADA) uses a very strict blood collection procedure to set up the Athlete Biological Passport [11]. First, sampling should not occur within two hours after training or competition to avoid exercise induced plasma volume decrease and dehydration. Second, the athlete has to remain seated with both feet on the floor for at least 10 minutes before blood collection. Additionally, duration of tourniquet application should be minimized and should be released immediately after venipuncture is made [11], as prolonged tourniquet application is known to lead to an in increase [Hb] and Hct [12]. In research examining blood cell counts, a precise description of blood sampling procedures in the methods section is often lacking. However, a standardized protocol according to those guidelines and an accurate description in the methods section should be standard to help the reader to better interpret the data and compare data from different studies.

Despite the possible issues with plasma volume shifts, this study shows an acute increase in leucocytes following apnea, confirming the results of Bakovic et al. [6]. The increase in WBC is strong enough to be apparent despite probable posture induced plasma volume expansion and mainly consists of an increase in monocytes, lymphocytes and non-significant increase in neutrophils. This is in agreement with unpublished data from our lab (S1 File) which also show an increase in total WBC count consisting of increases in lymphocyte, monocyte and neutrophil count up to 10 minutes after a series of five maximal static seated breath-holds (Fig 1). Interestingly, Dolscheid-Pommerich et al. [13] also show a second increase in WBC 4 hours post apnea. This second-line increase in WBC cannot be ascribed to a decrease in plasma volume as only neutrophils increase, whereas other WBC types as well as RBC, remain constant. This second increase is confirmed by our unpublished data which show a second increase in WBC 2.5 and 3 hours post apnea, mainly driven by an increase in neutrophils (Fig 1C).

**Fig 1:**
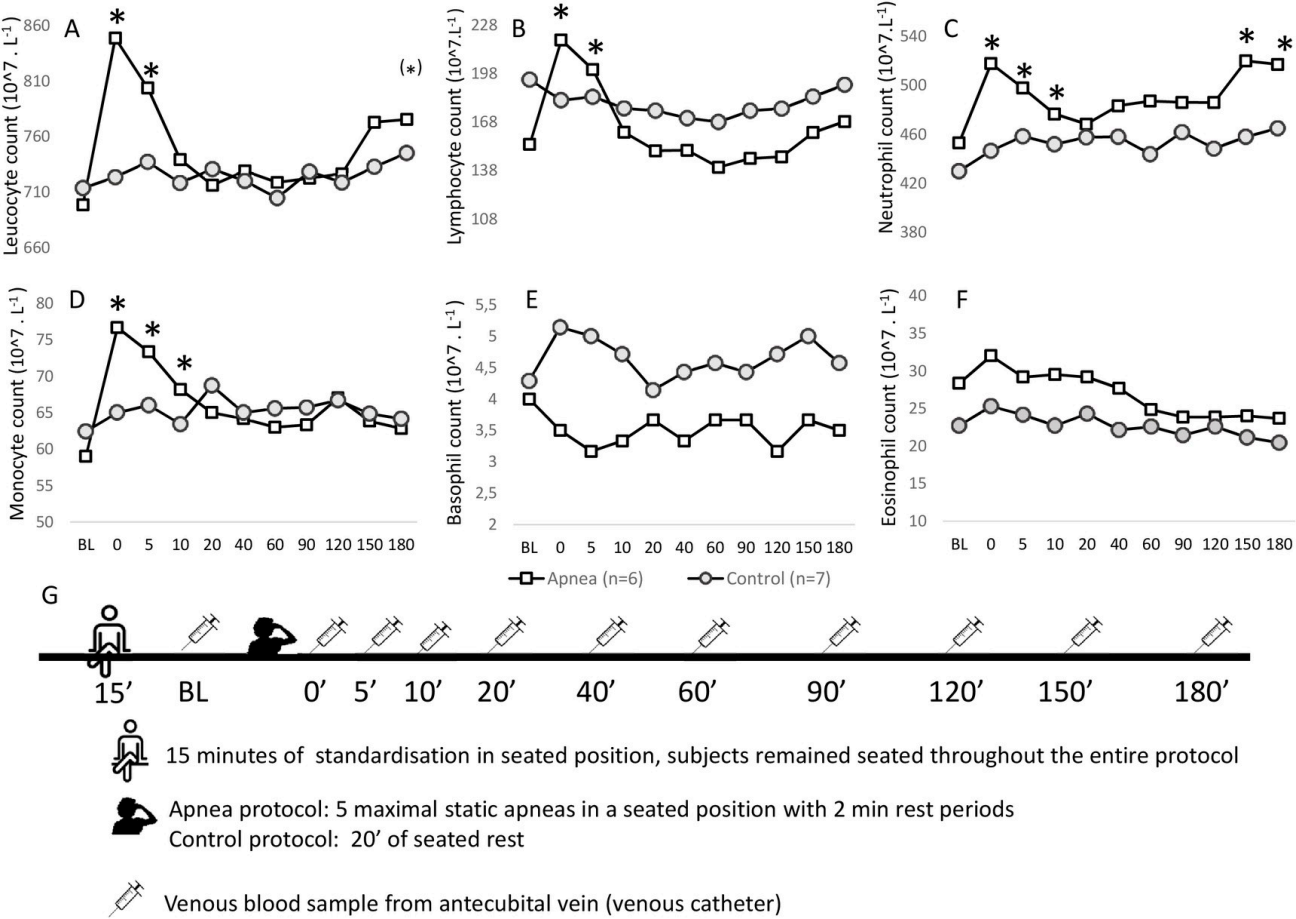
White blood cell response. Concentrations in cell counts are expressed as 10^7.L^-1^ for leucocyte (panel A), lymphocyte (panel B), neutrophil (panel C), monocyte (panel D), basophil (panel E) and eosinophil count (panel F) as measured by Sysmex XT2000i after 15 min of homogenization. Open squares represent the average of 6 subjects (n = 6) in the apnea condition (a series of 5 maximal seated apneas with 2’ rest intervals), the filled dots represent the average of 7 subjects (n = 7) for the control condition (15 min of seated rest). A visual overview of the protocol and methods is depicted in panel G. Venous blood samples (K3 EDTA 4 mL, Vacutest Kima, Arzergrande PD, Italy) were drawn from an antecubital vein before, immediately, 5, 10, 20, 40, 60, 90, 120, 150 and 180 min after a series of 5 static maximal breath-holds according to WADA guidelines [14]. All tests were performed at the same time of day and in a randomized order and at the same ambient air temperature (18°C) and humidity (48%). Subjects remained seated throughout both protocols to make sure that each blood sample was drawn after 15 min of seated rest. All protocols and procedures were approved by the local ethical committee of the Ghent University Hospital (EC UZG 2016/0033) and written informed consent was obtained from all subjects. * = statistically different from baseline (BL) at p < 0.05 in RM Manova pairwise comparisons. (*) = 0.05 < p < 0.10.

This double response pattern following apnea is especially interesting as both obviously have a different physiological origin. This biphasic response has already been established following catecholamine-administration [15] and was later confirmed in static breath-holding by Bakovic et al. [6]. While Benschop et al. [15] hypothesized that the first immediate increase was through β-adrenoreceptor stimulation, this was contradicted by Bakovic et al. [6] who suggested that the first immediate increase was caused by a spleen contraction, which is thought to be mediated through α-adrenoreceptor stimulation [16]. A spleen contraction induced increase is very plausible as lymphocytes are stored in the white pulp of the spleen and neutrophils and monocytes in the red pulp [17] matching the subtypes that increase in our data and those of Dolscheid-Pommerich et al. [13]. Alternatively, demargination of WBC due to an increased arterial blood pressure can also be hypothesized. An increase in shear stress within capillaries of structures holding marginated WBC might lead to a release of this WBC store into the circulating pool, similar to what is seen in acute exercise. Hemodynamic factors as well as increased catecholamine concentrations during exercise seem to be responsible for the majority of the WBC demargination [18]. In this context, the spleen has been suggested as a significant site of WBC demargination post exercise, while others disputed the extent of any spleen involvement [19]. However, the strongest argument for a spleen-induced increase immediately following apnea is the observation that the immediate increase was apparent in both trained apnea divers and healthy untrained subjects, while it was not visible in splenectomized subjects [6]. Additionally, increased WBC values return to baseline within 10 minutes which is consistent with the red blood cell response and corresponds to the time needed for the spleen to return to resting volume [7, 20].

The second ‘late’ increase in WBC is not likely to be induced by spleen contraction. First, as mentioned above, spleen volume returns to baseline within 10 minutes and our data do not show a second decrease in spleen volume in the period between 10 and 180 minutes post apnea. Second, although not statistically significant, the ‘late’ response was also observed in splenectomized subjects (+9.4%) [6]. The second increase could be an immunological response to the apnea-induced hypoxic stress similar to the acute phase immune response to exercise [21, 22]. This ‘late’ response was also seen in neutrophil count 4 hours following apnea diving sessions [23]. Sureda et al. [23] hypothesized that the exercise intensity of apnea diving alone was not sufficient to evoke a similar increase in neutrophils and suggested a combined effect of the exercise of apnea diving on the one hand and the hypoxia and reoxygenation on the other. Indeed, these new data confirm that static apnea increases neutrophil count in the hours following static breath-holding supporting the possible role of apnea-induced hypoxia in the increase in neutrophils. Although our data confirm the biphasic response of Bakovic et al. [6], the timing of the ‘late’ response is conflicting: Bakovic et al. [6] observed a second non-significant increase 20 and 40 minutes post apnea, while no increase was observed 30 minutes post apnea by Dolscheid-Pommerich et al. [13] and our data only show an increase from 150 minutes onwards. These recent data therefore suggest a later onset of the second response establishing at 2.5 hours post apnea and lasting till at least 4 hours post apnea.

In conclusion, the results of Dolscheid-Pommerich et al. [13] do not show an acute increase in red blood cells, contradicting a well-established apnea-induced response. We expect that this might be attributed to a change in plasma volume due to non-standardized blood sampling. We therefore advocate the need for a strict and standardized blood sampling procedure in scientific research. Despite this important methodological concern, the study reveals relevant insights concerning the response in a great amount of blood cell parameters in the hours following acute apnea. The biphasic response in white blood cells is especially striking as it not only confirms scarce previous evidence but also expands it with additional cell type measurements. These data appear to suggest a specific apnea-induced immune response in the hours post apnea.

## Supporting information

S1 File Raw data. This file shows raw data of the mentioned white blood cell parameters before and at 10 specific time points up to 3h after apnea and control.
(XLSX)
